# Composition and capacity of Institutional Review Boards, and challenges experienced by members in ethics review processes in Addis Ababa, Ethiopia: An exploratory qualitative study

**DOI:** 10.1111/dewb.12348

**Published:** 2022-03-05

**Authors:** Yemisrach Zewdie Seralegne, Cynthia Khamala Wangamati, Rosemarie D. L. C. Bernabe, Bobbie Farsides, Abraham Aseffa, Martha Zewdie

**Affiliations:** Armauer Hansen Research Institute (AHRI) working under the Clinical Trials Directorate; Center for Medical Ethics.; University of South-Eastern Norway, University of Oslo.; Brighton and Sussex Medical School, UK.; Armauer Hansen Research Institute.; Armauer Hansen Research Institute (AHRI) working on understanding Immune responses to Tuberculosis.

**Keywords:** capacity, challenges, Institutional Review Boards, resources, sub-Saharan Africa

## Abstract

Few studies in sub-Saharan Africa evaluate Institutional Review Boards (IRBs) capacity. The study aims to explore the composition of IRBs, training, and challenges experienced in the ethics review processes by members of research institutions and universities in Addis Ababa, Ethiopia. Our findings indicate that most IRBs members were trained on research ethics and good clinical practice. However, majority perceived the trainings as basic. IRB members faced several challenges including: investigators wanting rapid review; time pressure; investigators not following checklists; limited expertise in reviewing clinical trials, studies on genetics, and traditional medicine; lack of IRB offices for administrative work; competing tasks; limited staffing and the lack of a standardized review system. There is need for advanced training on research ethics to meet the evolving research needs. In addition, investments in IRBs are needed in terms of funding, and physical and human resources in Addis Ababa and Ethiopia in general.

## 1 ∣ INTRODUCTION

Ethics review of research by a competent and independent research ethics committee (REC) is imperative for protecting participants in biomedical research.^1^ In fact, the very mandate of RECs is to safeguard the rights, safety, welfare, and dignity of research participants.^2^

In reviewing research protocols, RECs should consider the following: (i) the benefits of the research in terms of the potential to improve health and generate new knowledge, (ii) scientific validity in terms of study design and methodology, (iii) fair selection of participants in line with the research objectives, (iv) benefit-risk balance, with benefits outweighing risks, (v) independence of the REC, (vi) informed consent with emphasis on sufficient knowledge and voluntary participation, (vii) respect for rights and well-being of the recruited participants and (viii) community engagement, among other things.^3,4^

Research indicates that there has been an increment in international collaborative health research in developing countries over the past few decades^5^ due to the high burden of infectious diseases.^6^ In addition, the globalisation of research and other more “practical” concerns such as lower costs, lesser regulatory demands, and availability of treatment-naïve patients have made industry and government sponsors from high income countries (HICs) to move clinical trials to low and middle income countries (LMICs) such as Asia, Latin America, and Africa.^7,8,9,10,11,12,13^ Consequently, there is an increased workload for RECs resulting from the high volume and complexity of biomedical research in LMIC.^14,15^ A considerable number of ethics experts and scholars have stated that the growth of biomedical research activities has not been complemented by a corresponding research ethics capacity enhancement in sub-Saharan Africa (SSA).^16,17^ A systematic review of 23 studies in SSA reported challenges hampering the effective functioning of RECs to include: lack of membership diversity, scarcity of resources, insufficient training of members, inadequate capacity to review and monitor studies, and lack of national ethics guidelines and accreditation.^18^ A small proportion of studies in SSA evaluate the capacity of RECs to perform ethics review. Thus, there is need for more studies as such evidence provides guidance for ethical review capacity-enhancement programs.^19^

## 2 ∣ ETHIOPIAN CONTEXT

Due to the various national and global efforts launched to understand and address the various diseases and search for therapeutic and preventative vaccines, there is an increase in Ethiopian biomedical research. In addition, the number of universities in the country have increased with expansion of graduate programs where research is a key component in the fulfillment of educational requirements. Along with the growth of research, there has also been a rise in awareness of research ethics and protection of research participants. Nonetheless, Ethiopia is at its infancy in terms of research ethics challenged by the diversity of culture and language and inadequate awareness of health research in the community.

In Ethiopia, there are RECs in research institutions and universities that are tasked with independently reviewing different types of research protocols involving human participants. There is also the National Research Ethics Review Committee (NRERC) that reviews sensitive research protocols such as those involving international collaboration, transfer of specimens abroad, and/or clinical trials.^20^ Clinical trials are also selectively reviewed by the Ethiopian Food and Drug Authority (EFDA). In addition, the Ethiopian Institute of Biodiversity provides authorization for the transfer of specimens and materials derived from plants, animals, or the environment abroad.^21^

Institutional Review Boards (IRBs) are constituted of medical and non-medical members, and their aim is to ensure the protection of the rights, safety and well-being of human participants involved in research and provide public assurance of that protection by reviewing the study protocols, the suitability of the investigator (s), facilities, and the proposed methods and material to be used in obtaining and documenting informed consent of research participants.

There is no public database to show formal registration of RECs in Ethiopia by the Ministry of Science and Higher Education and to indicate assessment of their composition, structure, and operational guidelines to confirm their adherence to national and international regulations with the exception of three RECs that were evaluated and recognized by the SIDCER (Strategic Initiative for Developing Capacity in Ethical Review) program of the WHO/TDR with the support of the regional FERCAP (Forum for Ethical Review Committees in the Asian and Western Pacific Region) and PABIN (Pan-African Bioethics Initiative). Nonetheless, the PABIN and ETBIN (Ethiopian Bioethics Initiative) have done various activities in the past to provide training to different RECs in East Africa, Ethiopia included.

An Ethiopian study that reviewed published and unpublished documents on research ethics from the Addis Ababa University-College of Health Sciences (AAU-CHS) from 2007 to 2012 identified several gaps and challenges of IRBs. The gaps and challenges included the need for refresher trainings at a regular interval, adequate supply of resources (office, equipment, and finance), inability to compensate members for their time and effort, and lack of follow-up of approved proposals due to budgetary constraints and poor compliance from researchers.^22^

Several years have passed since the last study was conducted. As such, there is need for more research to increase knowledge on gaps and challenges with regards to the capacity and functioning of RECs in Ethiopia. This study aims to explore the composition of IRBs, training and training needs, and challenges experienced in the ethics review processes by IRB members of research institutions and universities in Addis Ababa, Ethiopia.

## 3 ∣ METHODS

### 3.1 ∣ Study design

The study employed an exploratory qualitative design that entailed in-depth interviews with IRB members in Addis Ababa.

### 3.2 ∣ Study area

The study was conducted in Addis Ababa. Addis Ababa is the capital and largest city in the country of Ethiopia. Located in the center of Ethiopia, Addis Ababa currently has a population of 4.8 million people in the urban area and 2.7 million people in its city area.^23^

The studied IRBs were selected because they are part of national institutions with a long history of biomedical research and have experienced senior members who can independently review different types of research protocols involving human participants and are authorized to give ethical clearance by the NRERC. They also possess a wealth of experience that is resourceful to the study.

### 3.3 ∣ Sample and sampling strategy

Ten IRB members were included in this study. Table 1 lists the research participants based on the organization they work in, sex, and years of experience as IRB members. Participants are members of four IRBs: two IRBs from research institutions and two from universities. Purposive sampling was used to recruit the research participants; research participants had to be active members of the IRBs. We ensured that the IRB chairperson, IRB secretary, nonmedical person and medical professional were part of this study so that different professional backgrounds were represented.

### 3.4 ∣ Data collection

The data collection process took place from July to December 2018. The first author conducted the semi-structured in-depth interviews. The audio-recorded interviews took place in private spaces at the workplace of the participants. They were conducted in Amharic lasting approximately 30-45 minutes. They were later transcribed verbatim by the first author. The transcripts were then translated to English by a consultant with a qualification in social science and behavioral studies. A semi-structured interview guide was used in the discussions. Themes explored were structure and composition of IRBs, training, ethics review processes, and challenges experienced.

### 3.5 ∣ Data management and analysis

Thematic inductive data analysis was conducted by identifying, analyzing, and reporting patterns within the data.^24^

The iterative analysis entailed (i) familiarization with the data, (ii) generation of initial codes, (iii) search for themes, (iv) review of themes, and (v) defining and naming of themes.^25^ After data collection, transcripts and field notes were read and reread several times in order to gain an in depth understanding of the data^26^ and coded manually by the first and second authors. The authors then developed a table of subthemes which were reviewed and deliberated upon amongst themselves. Four themes were agreed upon: (i) structure and composition of IRBs, (ii) training, (iii) ethics review processes, and (iv) challenges.

### 3.6 ∣ Ethical considerations

The study was approved by the AHRI/ALERT (Armauer Hansen Research Institute)/(All African Leprosy, Tuberculosis and Rehabilitation Training Center) with the Ethical clearance no of PO35/18. Informed consent was sought from all the participants. Participants were informed that their participation was voluntary and assured of confidentiality and privacy of the shared information.

## 4 ∣ RESULTS

### 4.1 ∣ Structure and composition of IRBs

Of the four IRBs that participated in this study, three use a primary review system. A primary review system is a system where two reviewers review a research application to see if they meet the set criteria and make recommendations to the board whether the study should be approved or not. One IRB uses a secondary review system where the principal investigator provides a brief explanation of the study. The study protocols that do not fall in exempt or expedited review categories are then forwarded for full committee review. This is the most rigorous level of review and, accordingly, is reserved for research projects that present more than minimal risks to subjects.

Three of the IRBs (IRB 1, 3 and 4) use a permanent list of members while one (IRB 2) has two types of membership, namely permanent and alternate membership. In the latter IRB, permanent members review study proposals regularly, while alternate members are invited as a replacement in the absence of permanent members.

The number of members in these IRBs ranged from 9 to 18. Almost all members had undergone research ethics and good clinical practice (GCP) training. All members had formal education ranging from a bachelor's degree to post-doctoral training in either in Science or Social Studies. The experience of participants in serving the IRBs varied: 8 participants had served their IRBs for more than two years while 2 participants had served for a period of one year (see Table 2).

### 4.2 ∣ Training

From the study findings, most of the research participants had attended trainings that focused on fundamentals of research ethics (10 participants) and GCP (6 participants). Few participants had attended trainings that focused on consent processes (2), IRB standard operating procedures (2), data safety and monitoring board (DSMB) functions (1), research integrity (1) and biomedical ethics (3).

When asked about the kind of training and when it was attended, two research participants stated:

I attended GCP 2 days training, DSMB (clinical trial) and research ethics 3 days training…. I took GCP and research ethics training in 2014…

(R02, 9 years' experience, Academic IRB member)

I attended GCP, research ethics and different trainings… on clinical trials and other components…I took them 4 years ago.

(R09, 4 years' experience, Research IRB member)

Although 6 out of the 10 participants had received their training within the past 2-8 months, the time elapsed since the last training ranged from two weeks to five years. According to the participants, trainings were useful on equipping them with knowledge on research ethics principles and the review of research protocols and providing a platform for sharing experiences.

Two members said:

The trainings were useful especially on ethical principles and ethical meanings, and making ethical principles practical….systems and approaches of reviewing of different study protocols and sharing of experiences between IRB members.

(R06, 7 years' experience, Research IRB member)

They are very helpful, I joined this unit as a researcher and (the training) guides me on what parts of the protocol I should focus on (approaches to review a proposal), basic research ethics and its principles.

(R07, 31/2 years' experience, Research IRB member)

Nine out of ten IRB members indicated that the trainings were basic and recommended that future trainings focus on advanced research ethics, informed consent in relation to minors (children and adolescents), training others on research ethics, ethical issues in reviewing clinical and genetic studies and studies on traditional medicines, conflict resolution in the review process, and monitoring of studies after ethics approval.

Three participants explained:

I want to attend trainings that are helpful in building my capacity in my reviewing process, advance research ethics in depth and to organize and conduct such trainings for other researchers will also be very helpful.

(R07, 31/2 years' experience, Research IRB member)

I think trainings should be conducted regularly, there is progress in the scientific knowledge and diseases too. I want to take trainings on genetic studies, clinical trials and traditional medicine. Sometimes I read study proposals and we took this amount of blood and I confronted with questions on what the risk will be.

(R09, 5 years' experience, Research IRB member)

Yes, basic trainings are helpful to review a proposal as an IRB member, but actual reviewing process in training is necessary, how it will be handled if there is conflict between IRB members. It is also important to be trained on efficiently monitoring mechanisms after giving an ethical approval to confirm their practicability, keeping study participants safe, conducting ethical committee meetings and the reviewing process

(R02, 9 years' experience, Academic IRB member)

One member recommended that they should visit other IRBs and observe their practices as part of learning.

The participant said:

I would like to attend other IRB reviewing process to use it as a benchmarking experience, other IRB management especially the secretariat contribution.

(R03, 6 years' experience, Academic IRB member)

### 4.3 ∣ Ethics review processes

When asked about the types of protocols reviewed, the IRB indicated that different designs of research protocols were reviewed including longitudinal, cohort, cross-sectional, case studies, hospital and community-based studies, health related surveys, epidemiological, social, pre-clinical and laboratory related research protocols. They also indicated that they reviewed project-based studies and studies conducted by institutions such as public health service program evaluation, surveillance studies, diagnostic evaluation of medical device and social science studies.

Two members stated:

Basic research, clinical (prospective), multisite crosssectional surveys and sometimes genetic and clinical trials which are referred to NERC for ethical approval confirmation.

(R03, 6 years' experience, Academic IRB member)

…. epidemiological, social preclinical and laboratory related research.

(R04, 10 years' experience, Research IRB member)

More frequently our unit reviews students' proposal (Masters and PhD) and project-based studies.

(R05, 1 year experience, Academic IRB member)

Our institute works on different studies. We frequently review studies conducted by the institute such as public health service programmatic evaluation studies, surveillance studies, diagnostic evaluation of medical devices and basic research.

(R06, 7 years' experience, Research IRB member)

Regarding the review process, participants were asked to list parts of the protocol they gave emphasis to and the associated reasons. Most of the participants replied that their IRBs mainly focus on methodological and ethical issues such as consent, privacy and confidentiality, rights of study participants, benefits of the study to the study participants and the community, risks, and issues of justice.

Participants explained:

We try our best to review all parts of the protocol to check if the study is redundant or not, the methodological part, information sheet, consent, the risks, confidentiality, to maintain autonomy, ensure beneficence and protect the study participants from research risk.

(R01, 2years and 4 months' experience, Academic IRB member)

Methodology part, scientific component, ethics, consent, applicant qualification.

(R03, 6 years' experience, Academic IRB member)

I give more emphasis to the ethical parts of the protocol such as contents of the informed consent sheet like the confidentiality, justice, risk and beneficence part and the right to decide to participate in the study. I also evaluate the study in terms of scientific soundness.

(R06, 7 years' experience, Research IRB member)

Participants were asked about how they handle research protocols that were outside their expertise. They said that they sought help from other parties such as consults, peers, and literature or would refer to other institutions as indicated by Table 3.

### 4.4 ∣ Challenges

Participants were asked about the challenges they experienced in the ethical review processes. Some of the participants reported the need for rapid review and approval by study investigators.

Participants explained:

There is urgency to have ethical approval after submitting in a short period of time. There are some projects that were started in other Africa countries and because of this they urge the IRB unit to give ethical approval as soon as possible.

(R04)

Pressure from researchers, researchers have an interest to have their ethical approval letter in the next day of submission of their proposal.

(R08)

The participants said that the pressure also increased in cases of epidemics where they indicated that the study proposals needed to be reviewed as soon as possible to reduce morbidity and mortality rates. Some of the participants found it hard to review the research protocols on time as they had other responsibilities besides ethics review. A participant said:

Time is a challenge, IRB work is not a full time position and we do that in addition to other responsibilities.

(R08)

Another challenge was investigators not submitting required documentation for ethics approval. A participant stated:

Some research protocols do not fulfill the requirements such as IRB check list, collaborative approval letter, signature of all investigators, CVs and that is why the protocols were not reviewed because of the said gaps.

(R08)

Some of the members lacked expertise on certain aspects. One participant said:

I have limited knowledge and expertise to review, evaluate and gave ethical approval for medical equipment, clinical trial studies and genetic studies.

(R07 Research IRB member)

Administrative challenges were also raised. The participants argued that not having a permanent and functioning IRB office was negatively affecting their independence. A participant said:

Ethical review office should have a permanent office to confirm the unit's independence, quality in terms of training and knowledge, dedicated working staff, composition in terms of working procedure & process, using fixed evaluation system and practicing procedures and principles.

(R06, 7 years' experience, Research IRB member)

Non-scientific IRB members had not been trained on the review process and as such could not contribute fully to the process. The participant stated:

Sometimes nonscientific members of IRB are invited in the review process for fulfilling the criteria (presence of community representative). It would be better to support them by training to maximize their contribution in the ethical reviewing process.

(R06, 7 years' experience, Research IRB member)

## 5 ∣ DISCUSSION

This study explored composition of IRBs, training and training needs, the ethics review process, and challenges experienced by IRBs members in Addis Ababa, Ethiopia.

Three of the IRBs (IRB 1, 3 and 4) use a permanent list of members while one (IRB 2) has two types of memberships, namely permanent and alternate membership. All members had formal education ranging from a bachelor's degree to post-doctoral training in either in Science or Social Studies. Despite diversity in membership, the IRB in our study lacked members with previous expertise and experience in clinical trials to help review the submitted clinical trial documents accordingly. In addition, there was a lack of lay community representation to reflect social and cultural diversity and inclusivity of the community in research that affects them. Similar findings have been reported elsewhere.

The core of an IRB and its functions lie in the composition and expertise of its members. Composition must be multidisciplinary and should be able to reflect the social and cultural diversity of the communities from which the research participants are drawn; lay people and other members whose primary background is not in health research must be reasonably represented in ethics review processes.^27^

Most of the IRB members were trained on research ethics and GCP. However, majority of the members perceived the trainings to be basic and stated that they would like more training on advanced research ethics, informed consent especially when dealing with minors, trainer of trainers, and ethical issues in reviewing clinical and genetic studies, and traditional medicines. Similar findings are reported elsewhere.^28,29^ Some participants wanted to visit and participate in other IRBs processes as a form of learning/training. Most training of IRBs/RECs tends to be in workshops or structured courses;^30^ however, visits to other settings on ethics review processes would generate constructive criticism, reflection on processes and learning. Ethics training in similar settings should be cognizant of the fact research needs change and such frequent evaluations on training needs should be conducted and trainings tailored to meet those needs.

From our findings, the IRB members focused on methodological and ethical issues when reviewing research protocols. Regarding handling of research protocols outside their area of expertise, IRB members stated that they sought help from consultants, peers, read literature on the topic and at times would forward the protocols for review by other agencies.

Some of the challenges reported in the review process were investigators wanting rapid review, pressure to review research protocols on relevant epidemics and pandemics, investigators not following checklists and meeting requirements, lack of expertise in reviewing clinical trials, studies on genetics and traditional medicine, lack of IRB offices for administrative work, competing tasks, few staff members and a standardized review system. Studies elsewhere have reported RECs capacity to review clinical trials.^31,32,33^ A systematic review of 23 studies in SSA reported challenges hampering the effective functioning of RECs reported scarcity of resources, insufficient training of members, and inadequate capacity to review and monitor studies.^34^ The findings also align with studies elsewhere.^35,36,37^ Kithinji and Ikingura report that many African RECs lack institutional goodwill as ethical review processes are considered to be less taxing than developing proposals for funding.^38^ In addition, REC secretariats are understaffed and burdened with administrative work leaving less time on the main review processes.^39^ There is need for funding to support administrative duties of IRBs in Ethiopia to facilitate ethics review processes.

### 5.1 ∣ Limitation

This study is not without limitations. Being qualitative, the research findings cannot to be generalized. However, findings are useful for contexts with similar settings as Addis Ababa.

## 6 ∣ CONCLUSION

This study explored composition of IRBs, training and training needs, the ethics review process, and challenges experienced by IRBs members in Addis Ababa, Ethiopia. Findings indicate a need for advanced training on research ethics bearing in mind different study designs, topics and the challenges presented.

Advanced training on research ethics should include theoretical and practical sessions with experience sharing from certified IRBs. Trainees should be exposed to how an ethical review meeting is conducted as well as be given the opportunity for a guided review of studies based on the theoretical and practical exposure.

Western IRBs are usually trained with advanced research ethics courses; however, considering that not all Ethiopian IRB members would have access to scholarships for attending such training, it will be prudent to train IRB members locally. This will increase their knowledge and maximize the input of IRB members in the review process.

There is also need for funding to increase physical and human resources to facilitate ethics review processes. Training of and investment in RECs results in improved participants' knowledge of the ethical principles relevant to biomedical research, how effective REC should function and ensuring that studies are conducted safely and ethically.^40^

## Figures and Tables

**TABLE 1:** Sex, years of experience and type of institution

Participant	Sex	Years of experience	Research IRB member	Academic IRB member
R01	M	2 1/3		Yes
R02	M	9		Yes
R03	M	6		Yes
R04	M	10	Yes	
R05	F	1		Yes
R06	M	7	Yes	
R07	M	3 ½	Yes	
R08	M	2	Yes	
R09	F	5	Yes	
R10	F	1	Yes	

**TABLE 2:** Composition of RECs and study participants

	IRB1	IRB2	IRB3	IRB4	Participants
Number of members	11	18	9	10	10
SEX (% Male)	37.5%	72%	66.7%	50%	70%
**Member's education**					
BA/BSc	0	3	0	0	1
MSc/MPH	4	7	4	0	4
MD or (DVM)	5	2	3	5	3
PhD	1	5	2	4	1
LLB	1	1	0	1	1
**Members' profession**					
Social sciences	4	4	0	1	3
Science/Medical	7	14	9	9	7
Participants	3	3	3	1	10

**TABLE 3:** Managing mechanisms of a new type of proposal by IRB study participants

Actions taken	Number of participants (out of 10)
• Use consultants	06
• Consult with peer members	05
• Read on the topic	02
• Forward the review process to another agency/institution	04
